# Olfactory Impact of Terpene Alcohol on Terpenes Aroma Expression in Chrysanthemum Essential Oils

**DOI:** 10.3390/molecules23112803

**Published:** 2018-10-29

**Authors:** Yunwei Niu, Xiaoxin Sun, Zuobing Xiao, Pinpin Wang, Ruolin Wang

**Affiliations:** School of Perfume and Aroma Technology, Shanghai Institute of Technology, 100 Haiquan Road, Shanghai 201418, China; nyw@sit.edu.cn (Y.N.); Phoebe1397@163.com (X.S.); wang_pinpin@163.com (P.W.); wangruolin82@163.com (R.W.)

**Keywords:** *Chrysanthemum* essential oils, perceptual interactions, aromatic recombinations, synergy effect

## Abstract

The key point of our work was evaluating the impact of terpene alcohols on the aroma expression of terpenes recombination in Chrysanthemum essential oils. Using pure commercial products, various aromatic recombinations were prepared, consisting of terpenes recombination and six terpene alcohols, all the concentrations found in Chrysanthemum essential oils. There were five groups of terpene alcohols mixtures performed very interesting with the addition or omission tests. The “olfactory threshold” of the terpenes recombination had a notable decrease when adding isoborneol, d-Fenchyl alcohol respectively through the Feller’s additive model analysis. Furthermore, the descriptive test indicated that the addition of terpene alcohols mixture had the different effect on fruity, floral, woody, green, and herbal aroma intensity. Specifically, when isoborneol was added to the terpenes recombination in squalane solution, it was revealed that isoborneol had a synergy impact on herbal and green notes of the terpenes recombination and masked the fruity note.

## 1. Introduction

Chrysanthemums are of the genus (*Chrysanthemum*), belonging to the Asteraceae family which is very common in China [1]. Additionally, *Chrysanthemum* essential oils are widely used in food industry [2,3,4]. There are many attributes to identify the quality of chrysanthemum, where the mainly outward appearance, color and aroma [5]. Aroma characteristics have contributed immensely to the value and appeal of many food products, and have largely determined what consumers are willing to pay for many food products. Meanwhile, Chrysanthemum essential oils were well-known by its representative aroma. However, researchers were inclined to study the chemical composition, extraction method and antibacterial activity of chrysanthemum essential oils [6,7,8,9,10,11,12], the researches on the sensory effects of volatile compounds of chrysanthemum essential oils was lacking in-depth exploration.

The volatile constituents of chrysanthemum essential oils are mainly terpenes, terpeneesters, terpene aldehydes, aliphaticacids, and terpene alcohols. In terms of quantitative point of view, terpenes and terpene alcohols are the primary components in Chrysanthemum essential oils. Although chrysanthemum aroma is a complex mixture of various components, terpenes usually are the dominant in the volatile profile [4,13,14]. At the same time, terpene alcohols asthe relatively important part of aroma contribution in chrysanthemum essential oils have not been reported systematically so far [15]. While the existence of them were already known, the effects on sensory perception have not been studied in detail. Therefore, in this paper, we focus on the aroma interaction between terpenes and terpene alcohols.

Odor perception is one of the foremost criteria used by the consumer to assess the quality of chrysanthemum essential oils. However, the complexity of odor perception, especially the perception of complex odor mixtures, makes it difficult to find the disciplinarian of aroma changes. Some fundamental rules have been found in binary and more complex mixtures concerning perceptual interaction [16,17,18]. These fundamental rules are very helpful for us to learn the aroma interaction better. Adhikari et al. [16] measured the olfactory threshold of diacetyl, hexanal and decalactone and found that adding hexanal to diacetyl in water repressed aroma emission of diacetyl, but adding hexanal to decalactone accelerated the emission of decalactone. Cain [17] has researched the mixtures of five concentrations (0.004–0.88 mg/L) of pyridine and linalyl acetate (0.09 mg/L) and five concentrations (0.004–0.88 mg/L) and linalool (0.03 mg/L) and found the lowest concentration of pyridine in the mixture was masked completely. Ferreira et al. [18] summarizes the results of 35 different mixtures containing different proportions of pyridine and butanol and found that most of these mixtures follow a partial addition behavior.

Therefore, the aims of the present study were researching aroma expression of chrysanthemum essential oils by (a) testing the steadiness of the samples constitution in the mixture of terpene alcohols and terpenes recombination; (b) selecting the major aromatic terpene alcohols by addition or omission tests; (c) investigating sensory effect of terpene alcohols on quantitative perception; and (d) using multivariate statistical analysis and electronic nose technology to research the effect of terpene alcohols addition on TR sensory properties. Through the above research, we will improve the understanding of the aroma interactions in Chrysanthemum essential oils and compensate the typical aroma compounds to a better characteristic aroma of chrysanthemum.

## 2. Materials and Methods

### 2.1. Chemicals

α-pinene, isoborneol, 4-terpineol, β-pinene, camphene, caryophyllene, β-myrcene, d-fenchyl alcohol, α-phellandrene, eudesmol, germacrene B, dl-limonene, cis-ocimene, α-terpinolen, β-farnesene, linalool, borneol were purchased from Shanghai Titan Technology Co., Ltd. (Shanghai, China). A homologues series of alkanes (C7–C30) was provided by Sigma–Aldrich Chemical Co. (St. Louis, MO, USA). Squalane was from BASF Co. Ltd. (Shanghai, China).

### 2.2. Aromatic Recombination

The terpenes recombination (TR) was prepared with 11 terpenes in alkane solution at the average concentration found in Chrysanthemum essential oils. Alkanes are colorless and odorless and alkanes total had 17–25% in Chrysanthemum essential oils. In this experiment, squalane with a similar chemical structure was used as the mixed alkanes substitute. Squalane colorless and tasteless, and safer, non-toxic, economical, can be used for subsequent food addition applications [19,20]. According to Xiao et al. [21], the concentration of each compound in complete aromatic recombination is shown in Table 1. The three levels concentrations of 5 terpene alcohols were listed in Table 2.

### 2.3. Terpenes and Terpene Alcohols Analyses

#### 2.3.1. Solid Phase Micro Extraction (SPME) Condition

The manual SPME holder, together with 20 mL vials, Teflon covers and one fiber (50/30 μm DVB/CAR/PDMS) purchased from Supelco, Inc. (Bellefonte, PA, USA). The fiber was pre-conditioned on an Agilent 7890 gas chromatograph (Agilent Technologies, Santa Clara, CA, USA) at the injector temperature of 250 °C. Injection of SPME fibers was carried out at a split ratio of 1:25. During the SPME experiment, we set the extraction temperature at 25 °C. Five ml of samples were transferred to the vial and seal it hermetically with the Teflon lid. The vial was held on a SPME heater at 25 °C at a stirring speed of 80 rpm for 40 min. After exposing the SPME fibers to the upper space of the vial with 40 min, then transferring the fiber to the injector for desorption at 250 °C for 5 min. The experiment was performed in triplicate.

#### 2.3.2. Gas Chromatography−Mass Spectrometer Analyses

The aromatic recombinations were analyzed by Agilent 7890 gas chromatograph (GC) system coupled with a 5973C mass spectrometer (MS) equipped with an HP-Innowax column (60 m × 0.25 mm id, and 0.25 μm, film thickness; J&W Scientific, Folsom, CA, USA). The carrier gas was helium with 1.0 mL/min flow rates. The MS running data as follows: The ionization potential was 70 eV, ion source temperature was 230 °C and the mass range was 30–450 amu; oven temperature was 50 °C for 5 min, programmed rising to 230 °C by 3 °C/min with a 10 min holding time.

### 2.4. Electronic Nose

The volatile response change was monitored by electronic nose (Alpha M.O.S., Toulouse, France). The instrument loaded 18 sensors. The headspace bottles loaded 3 g liquid containing aroma recombination sample and sealed by a Teflon cover, then placed in an orderly manner in an autosampler. Each sample was hold in 60 °C for 6 min with agitating (500 rpm). The headspace was injected at a rate of 150 mL/min. Volume injected at every turn was 250 μL, and measured each sample in four replicates.

### 2.5. Olfactory Analyses

#### 2.5.1. Laboratorial Environment

The condition of sensory analyses was performed as described by Martin and de Revel [22]. Each sample was evaluated in a separate section using black ISO glass at room temperature [23]. Each meeting lasts about 5 min. A gap of 20 s was sufficient between individual odor assessments.

#### 2.5.2. Addition and Omission Test

Panel 1 consisted of 32 panelists, 15 males and 17 females, ages 25–39, average 27 years. All panelists learned about the aroma of Chrysanthemum. Panelists need to participate in three training a week, and each session lasts 5 min for a total of 4 weeks. In training sessions everyone was familiarized with the samples to be consumed in the test. The order of presentation throughout the session was irregular with respect to concentration and odor quality. Panelists were not told, nor could they tell by visual cues, whether the vessel they received on any trial contained aromatic recombinations or alkane solution alone. The order of presentation throughout the session was irregular with respect to concentration and odor quality.

The aroma recombination samples were estimated using triangular test method by panel 1 [22] (Table 3 and Table 4). A first group of triangular tests for addition (Table 3; test 1–26) included the evaluation of the perception of each set of terpene alcohols in the terpenes recombination. The concentration of each terpene alcohol in aromatic recombinations was listed in Table 1. In the second phase, triangular omission tests were performed on the same panel (Table 4; test 27−58). Series were presented in descending order of concentration. In each sets, demands that the panelists smelt three samples orthogonally from left to right and gave an answer compulsively during the tests. For each triangular test, three numbered samples were presented in random order: Two identical and one different. The significance of the difference in test results according to the method proposed by Roessler [24].

#### 2.5.3. Olfactory Thresholds Test

Panel 1 determined the olfactory thresholds of d-fenchyl alcohol (F), isoborneol (I), linalool (L), eudesmol (E), borneol (B), mixture of borneol and eudesmol (BE), mixture of borneol and linalool (BL), mixture of eudesmol and linalool (EL), mixture of borneol, eudesmol and linalool (BEL), and TR by three-alternative forced choice method. The olfactory thresholds of the mixtures of TR and F, I, BE, BL, EL, and BEL were measured. Each session was diluted to 10 gradients with a multiple of 2.

The results were statistically analyzed by Feller’s additive model. When the probability of detection was 50%, the detection threshold was defined as the concentration. This statistic was confirmed by adapting the ASTM-E1432 method [25]. The function used by Feller’s additive model accords with the sigmoid curve (y = 1/(1 + exp( − (x − C)/ D)), where C represents olfactory threshold of each aromatic component meant by log(mg/L), D represents the parameter of every aromatic component, x indicates vapor concentration meant by log(mg/L), and y indicates the detecting probability of odorant [26].The probability of detection is corrected by accidental factor formula P = (3·p − 1)/2, in which P refers to the corrected ratio of the accidental factor formula, and p refers to the actual detecting probability. All of the experiments were replicated in triplicate by panelists. Sigma Plot 12.0 (SYSTAT) software was used for graphic resolution.

Furthermore, Feller’s additive model, according to the report of Miyazawa et al., was used to evaluate the interaction effects of certain mixtures [27,28], the formula was used: P(AB) = P(A) + P(B) − P(A)P(B), in which definitions of P(A) and P(B) are the detecting probability of component A and component B alone, P(AB) refers to the detecting probability of mixture. According to the model, if the panel’s detection performance for the mixture matched response addition, then little or no mixture interaction had occurred. If performance falls above response addition, then some form of mutual enhancement or synergy had occurred. Moreover, if performance falls below response addition, some degree of suppression has occurred.

#### 2.5.4. Descriptive Testing Methods

Panel 2 was formed of 9 males and 9 females, aged 21–26, with an average of 23 years. They were all laboratory staff and they were aware of the nature of samples and objectives of the study. They were selected to participate in descriptive groups who demonstrated sufficient discernibility, repeatability, and consensus with the rest of the group for at least 80% of the descriptors in the ballot.

Panel 2 performed descriptive analysis of isoborneol (3065 mg/L), d-Fenchyl alcohol (1177 mg/L), the mixture of linalool and borneol (3157 mg/L), the mixture of borneol and eudesmol (1360 mg/L), the mixture of eudesmol and linalool (4151 mg/L) in alkane solution. Descriptive analyses of the mixture of borneol, eudesmol and linalool containing 0.183 mg/L of borneol, 2974 mg/L of linalool, and 1177 mg/L of eudesmol was performed by the same panel. In the first phase, TR was packed into brown glass bottles and assessed by panelists. Panelists discussed it three times, each time lasting 2 h. In the end, panelists agreed that five sensory attributes (floral, herbal, green, fruity, and woody) could be used to describe the aroma characteristics of TR. Each of characteristic aroma descriptor was determined by the mean value of three digits and presented within ten point scales. In the second phase, the perceived odor intensity of each of the six key terpene alcohol groups and the mixture overall perceived odor intensities of TR and terpene alcohols were evaluated by panelists. Subsequently, panelists evaluated aroma intensity of TR and terpene alcohols on five sensory descriptors. The result was presented on a 10-point scale, where 0 represented no odor intensity and 9 represented the maximum intensity. The experiment above would perform in triplicate. The formula [σ = f(τ)] for the aroma effect of binary mixture was mentioned by Patte and Laffort [29], the experimental data is shown on the graph. σ refers to the ratio between the aroma strength of mixture (I_mix_) after mixing and total amount of aroma intensity of the unmixed component (I_A_ + I_B_): σ = I_mix_/(I_A_ + I_B_). τ indicates the ratio of the aroma strength of unmixed component (A or B) and the sum of the aroma intensity of the unmixed component I_A_ + I_B_, τ_A_ = I_A_/(I_A_ + I_B_) or τ_B_ = I_B_/(I_A_ + I_B_). Thus, when σ = 1, there is complete intensity addition, when σ > 1, there is hyper-addition and when σ < 1, there is hypo-addition. Then use these two parameters to calculate the aroma intensity of overall aroma, woody, green, floral, fruity, and herbal notes. For each sample, the significance of the observed perceptual interaction was statistically tested by calculating the 95% confidence interval on the mean intensity of the panelists’ mean values (mean of the 3 repetitions) for both σ and τ. All statistical analyses were conducted using the Wilcoxon signed-rank statistical nonparametric test (XLSTAT software).

### 2.6. Statistical Analysis

Data from the olfactory analysis was submitted to analysis of variance (ANOVA) using SPSS v13.0 (SPSS Inc., Chicago, IL, USA). Partial least squares regression was used to explore the relationship between samples, sensory attributes, and the sensor response values through UNSCRAMBLER ver. 9.8 (CAMO ASA, Oslo, Norway). By applying PLSR analysis to standardized data, the importance of the sensor response values towards each attribute could be compared quantitatively based on regression coefficients and loading weights for each predictor or X-variable used in PLSR models. The statistically significant level was 5% (*p* < 0.05).

## 3. Results and Discussion

### 3.1. Evolution of Sample Composition during Sensory Analysis

**Figure 1:**
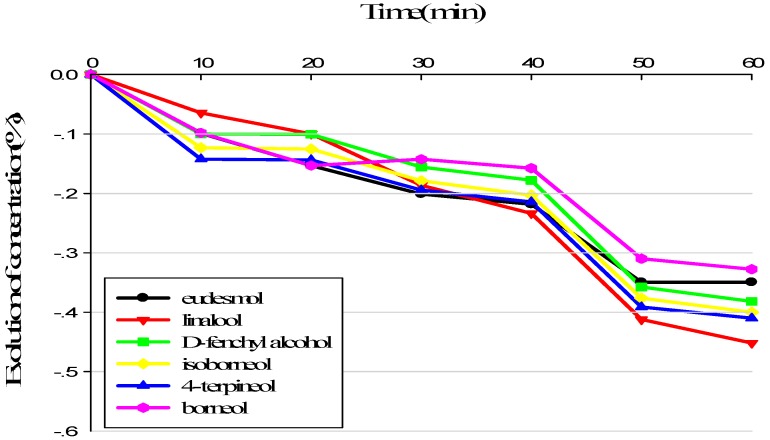
Volatie loss of terpene alcohols over time in sensory analysis in alkane solution.

**Figure 2:**
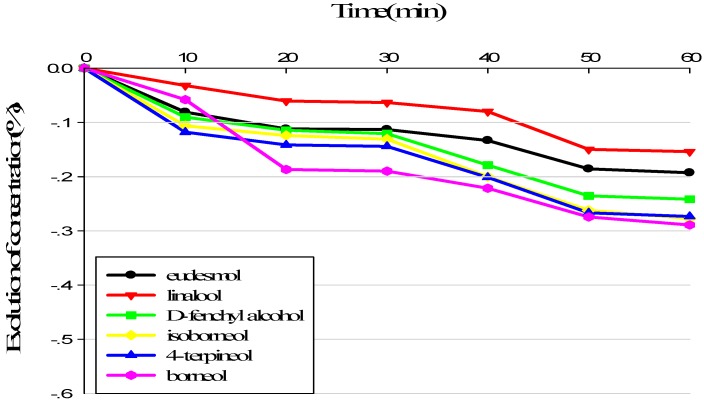
Volatie loss of terpene alcohols over time in sensory analysis in Terpenes Recombination.

The evolution of the terpene alcohols in alkane solution and in terpenes recombination (TR)was evaluated in order to assess the stability of the component of samples submitted to the panel. As can be found out from Figure 1, terpene alcohols kept steady in the alkane solution for 20 min. Afterward, the concentrations of the compounds in the solution decreased obviously. After 50 min, the concentrations of certain compounds in alkane solution had decreased by up to 40%. In TR, terpene alcohols kept stable for a longer period of time and lost no more than 30% after 60 min, with the exception of borneol (Figure 2). In TR, the terpene alcohols were more stable over time than in alkane solution. Consequently, the samples presented to the panel were prepared every 50 min. Georgia et al. [30,31] confirmed the better stability of demethyl sulfide in a complicated matrice than in a single matrice, and they observed that these compounds kept stable for about 10 min. He also evaluated the stability of 12 esters in wine or in aromatic reconstitution, and the results showed that the majority of esters kept more stable in wine.

Our results verified that the matrix affects the stability of aroma components. The aromatic evolution is due to physicochemical phenomena. The composition of the headspace changes over time as some compounds evaporate, according to their affinity for the matrix. The matrix was apparently able to modulate the composition of the headspace, thus impacting the aromatic perception of the sample [32]. The interaction may be affected by the physicochemical properties of the aromatic compounds, as well as van der Waals forces or hydrogen bonding.

### 3.2. Organoleptic Effect of Terpene Alcohols on Quantitative Perception

#### 3.2.1. Addition and Omission Tests

The addition of F, I, EL, BL, BE, BEL, LEFI, BELFI, BELFT, EFLIT, or LEBIT in TR was significantlyperceived (Table 3, test 2, 5, 7, 8, 16, 22, 23, 23, 25, and 26), the rest of the aromatic recombinations (the rest of tests shown in Table 3) were not significant in TR.

As listed in Table 4, samples lacking of isoborneol (Table 4, test 31, 36, 40, 43, 45, 47, 48, 50, 51, 52, and 54–58), the judges had easier differentiating the samples, and demonstrated isoborneol was important for the aromatic recombination. Moreover, EL, BL, BE, and BEL (Table 4: test 34, 35, 42 and 49) omissions were significantly perceived by the panel. Furthermore, when d-fenchyl alcohol was presented individually in TR (test 2), the judges had more difficulty differentiating the samples, compared to the complete recombination (test 28). This illustrates the impact of the aromatic complexity of the matrix on the detection of this molecule. The significant perception difference, probably due to the presence of the five other terpene alcohols, it may raise its perception threshold in such conditions. Thus, also considering the impact of the individual omissions and adding of these compounds, these results tend to highlight the important role of isoborneol, d-fenchyl alcohol, EL, BL, BE, and BEL in Chrysanthemum essential oil.

#### 3.2.2. Olfactory Threshold of Terpene Alcohols in Different Matrixes

These olfactory thresholds of terpene alcohols in alkane solution were shown in Table 5. The findings concerning the olfactory threshold of borneol in water reported in the literature: 0.14 mg/L [33]. In our work, the olfactory threshold of borneol in alkane solution is 2.03 mg/L. The olfactory threshold of linalool in water reported 0.001 mg/L [34]; the olfactory threshold of linalool in alkane solution is 3.85 mg/L. While the evaluation of olfactory threshold could be affected by lots of factors [34], the olfactory threshold determination in the literature mostly uses water as the solvent, ignores the function of non-volatile compounds on the thresholds of aromatic compounds [35]. The differences in olfactory threshold between the obtained results and literature data proofed the matrix affected the intensity of volatile aroma compounds. It’s obvious that the olfactory threshold of terpene alcohols in alkanes solution was higher than the thresholds in water. Similarly, comparing the thresholds of terpene alcohols in different matrices between alkane solution and TR, the olfactory threshold of isoborneol was 0.61 mg/L in alkane solution and 3.22 mg/L in TR, the threshold is increased by a factor of about five; the olfactory threshold of BEL was 0.87 mg/L in alkane solution and 1.80 mg/L in TR, the threshold is increased by a factor of about two. The threshold concentration in different matrixes is different, and the threshold concentration of compounds in a complex metric is higher.

The olfactory threshold of borneol and linalool in alkane solution is 2.03 mg/L and 3.85 mg/L, respectively; the olfactory threshold of the mixture (BL) in alkane solution is 0.05 mg/L. In alkanes solution, d-fenchyl alcohol added to TR led to a decrease in the “olfactory threshold” of the mixture (from 30.69 to 12.74 mg/L). The addition of d-fenchyl alcohol led to a 2.41-fold (*p* < 0.001) decrease. That interesting finding has also revealed the aromatic interaction mechanism. Therefore, in order to study these effects, further experiments were carried out by adding three different concentrations of aromatic compounds to the terpenes recombination.

#### 3.2.3. Sensory Effect of Terpene Alcohols on Aroma Expression

The aromatic strength of the mixture was not simply the totality of its component strength [36,37,38]. Perception depends not only on the concentration of the stimulus, but also on factors such as memory, mood, expectation, age, or cross-pattern interactions [39]. The researchers divided the interactions between aromatic components into four categories [40]. In order to proof if there were quantitative contributions of terpene alcohols was due to synergism, simple addition phenomenon, or a hyper-addition effect, we have evaluated the effect by comparing the measured threshold with theoretical threshold calculated on the basis of Feller’s additive model. Meanwhile, detection probability for isoborneol at high, medium, and low level in *Chrysanthemum* essential oils individually have been tested by Feller’s additive model. 

**Figure 3:**
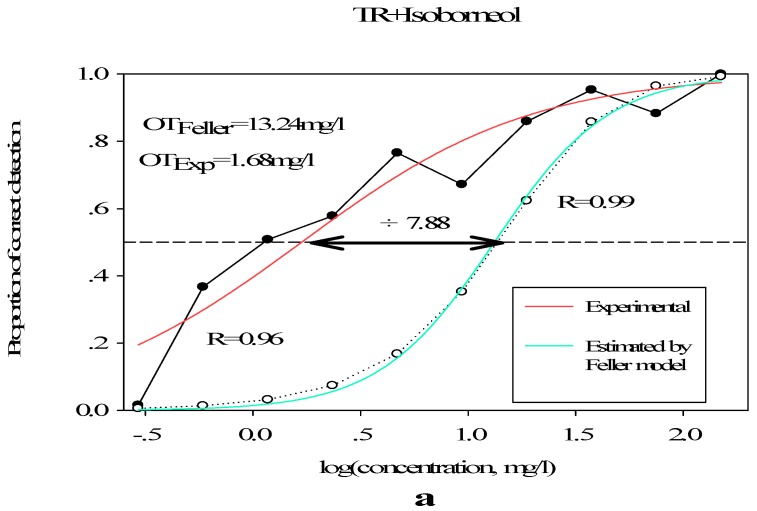

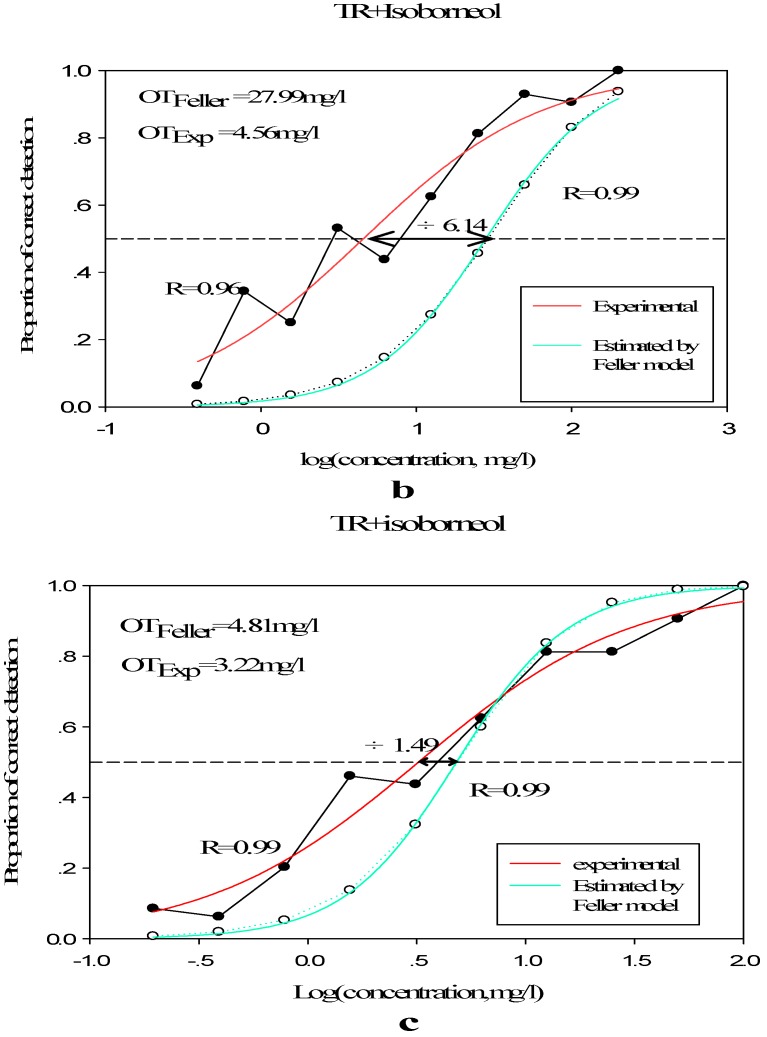
Comparison of detection probabilities of aromatic recombinations by experiments and by feller’s model. OT_Feller_, olfactory threshold measured by Feller’s additive model; OT_Exp_, experimental olfactory threshold. (**a**–**c**) Adding isoborneol at high, medium, and low level respectively.

The TR detecting probabilities in the presence of isoborneol with different concentrations (Figure 3) are higher than values tested by the Feller’s model. In the high concentration, the olfactory threshold measured by Feller’s additive model is 13.24 mg/L, the experimental olfactory threshold is 1.68 mg/L, the threshold is reduced by 7.88 times (*p* < 0.001), similarly, the threshold is reduced by 6.14 (*p* < 0.001) and 1.49 times (*p* < 0.001) at medium and low concentrations, revealing an addition effect when different concentrations of isoborneol were added to the TR. Depending on isoborneol addition tested, the addition of isoborneol had significant effects on the olfactory threshold of the mixture. This was consistent with the triangular test results.

According to the terpene alcohols tested in Table 5, the addition of BL, EL, or BEL led to a 9.53 (from 30.69 to 3.22 mg/L, *p* < 0.001)-, 1.42 (from 30.69 to 21.63 mg/L, *p* < 0.05)-, and 17.05 (from 30.69 to 1.80 mg/L, *p* < 0.001)-fold decrease, respectively, in the olfactory threshold of the TR, indicating a additive effect for each of these compounds in TR. And the addition of BE led to a 2.26 (from 30.69 to 69.34 mg/L, *p* < 0.001)-fold increase in the olfactory threshold of the TR, revealing a subtraction effect after the addition of BE to TR.

**Figure 4:**
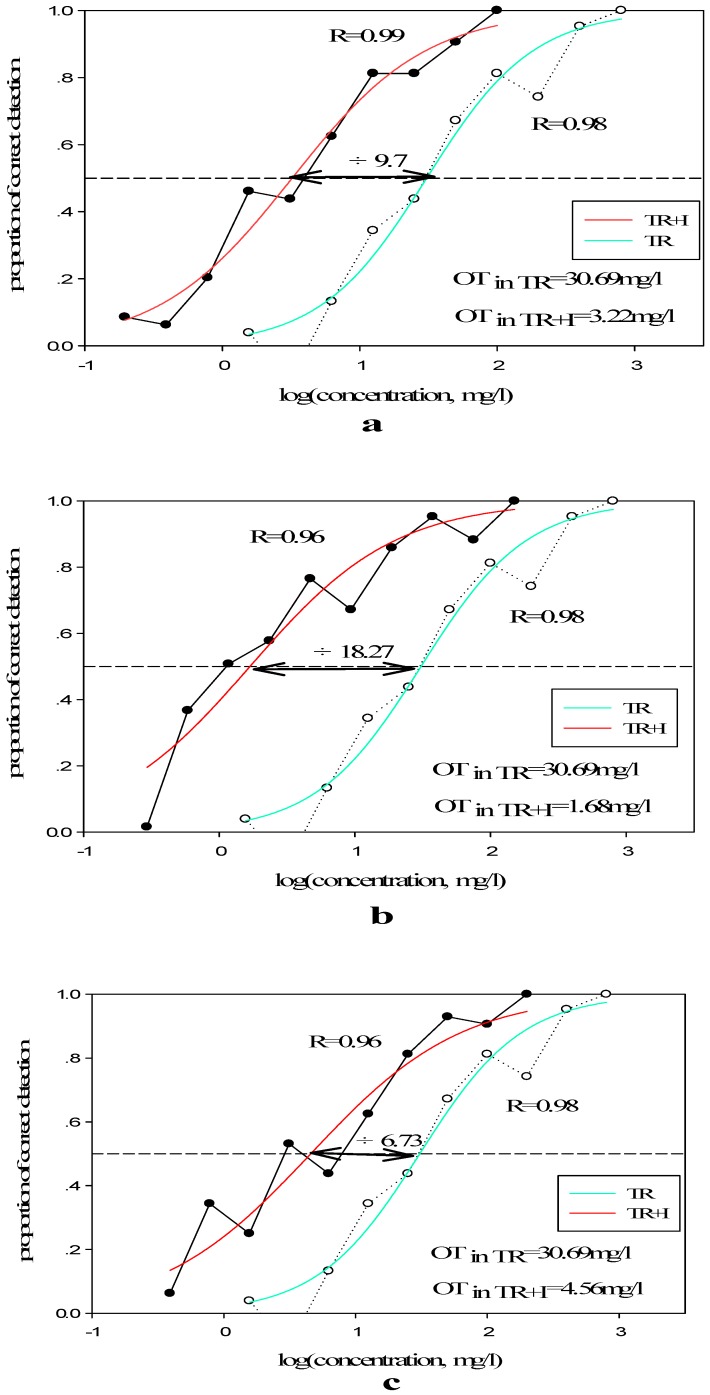
Aromatic effect of alcohols at different concentrations added to terpenes recombination. I, isoborneol. (**a**–**c**) Adding isoborneol at high, medium, and low level respectively.

Adding different concentrations of isoborneol led to a 9.70-fold at high level (*p* < 0.001), 18.27-fold at medium level (*p* < 0.001), and 6.73-fold at low level (*p* < 0.001) decrease (from 30.69 to 3.22 mg/L at high level, 1.68 mg/L at medium level and 4.56 mg/L at low level), respectively, and the result was showed at Figure 4. These results indicate that the addition of isoborneol leads to a decrease of TR olfactory threshold. Similarly, A De-La-Fuente-Blanco [41] found that ethyl phenol at concentrations of 365 and 375 mg/L increased animal aromas to varying degrees. In the wood wine model, subjects were able to detect aroma differences, even if the amount of higher alcohol added was trace, such as 17 mg/L and 22 mg/L. The perceptual interaction when Higher Alcohols were added to the Fruit Recombination was due to the complexity of the mixture, and devotion of a certain component to the overall aroma of aroma recombination is concerned with its concentration [42]. The presence of each compound resulted in a change in the olfactory threshold of the TR, reflecting an individual quantitative contribution of these compounds to overall aroma intensity.

### 3.3. Sensory Effect of Terpene Alcohols on Qualitative Aroma Perception

#### 3.3.1. Impact of the Addition of One or More Terpene Alcohols on Terpenes Recombination

Under the concentration shown in Table 2, a total of five descriptors were defined for terpenes recombination, namely floral, herbal, fruity, woody, and green. TR added isoborneol at different concentrations in alkanes solution resulted in several modifications.

**Figure 5:**
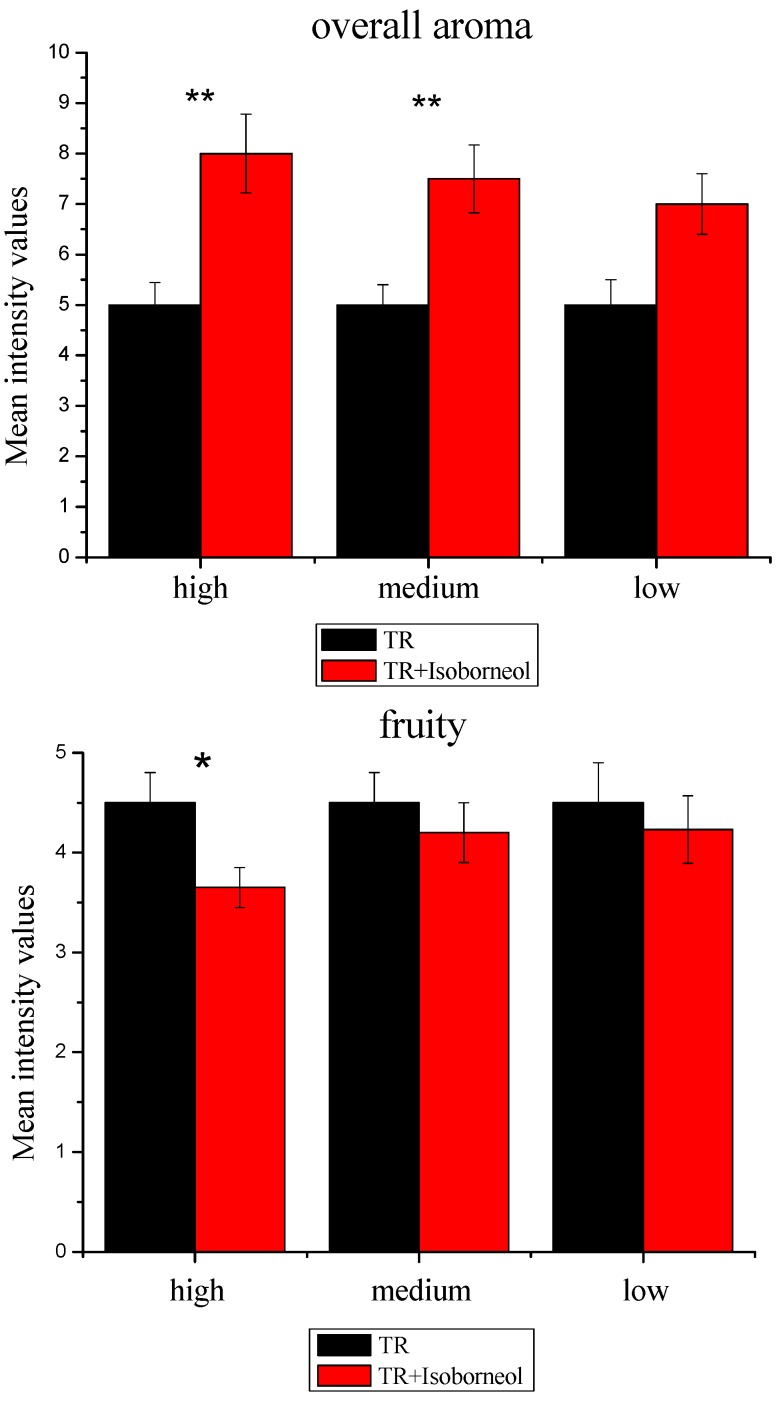

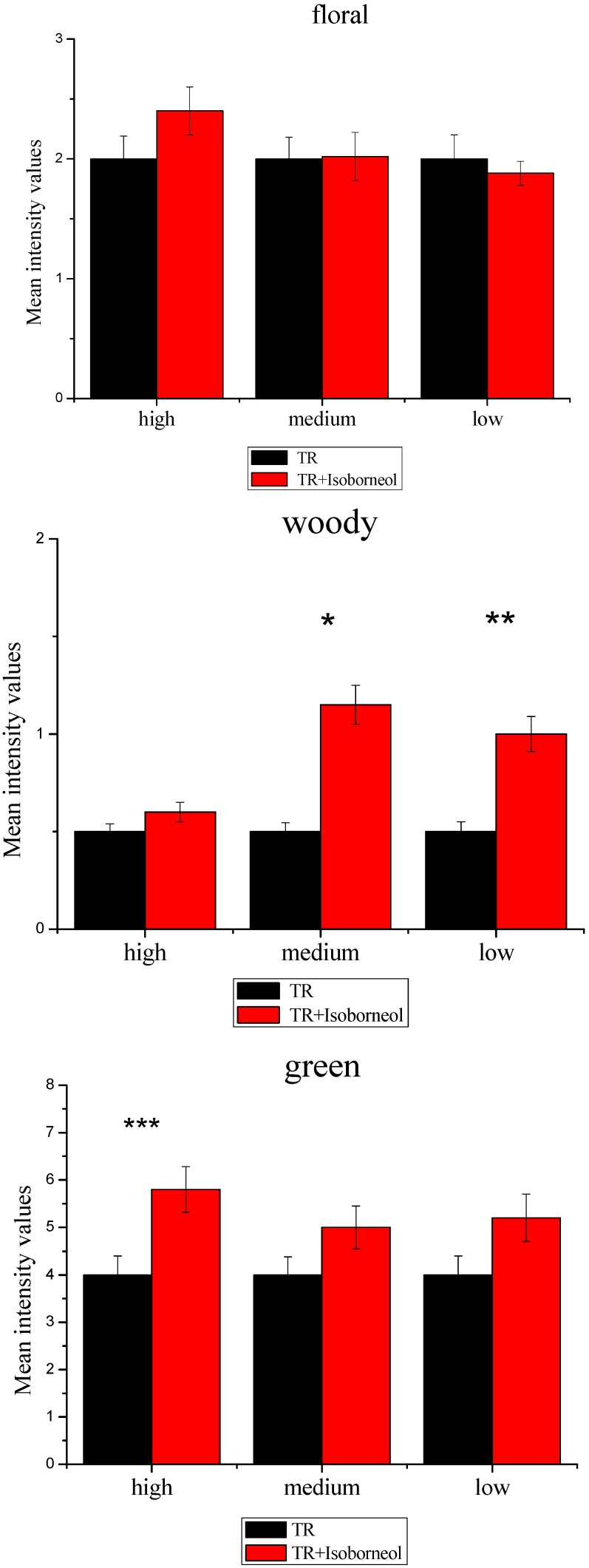

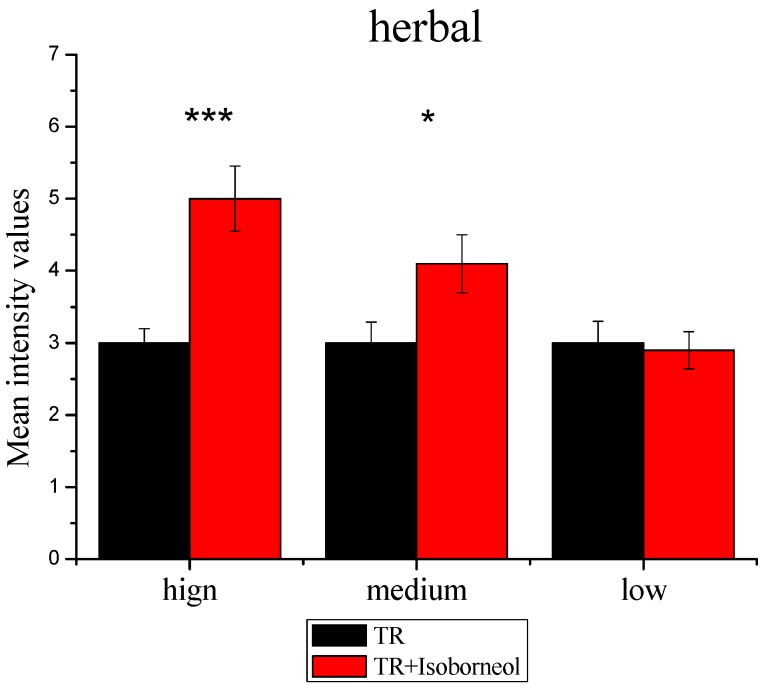
Aroma effect of three levels of isoborneol with terpenes recombination. *, *p* < 0.05; **, *p* < 0.01; ***, *p* < 0.001. TR, terpenes recombination. Error bars represent standard error deviation.

The variance analysis results showed that the adding high concentration of isoborneol caused a significant increase in the overall aroma (*p* < 0.01), this result was consistent with the olfactory threshold experiment (Figure 5). The addition of high concentrations of isoborneol (3065 mg/L) caused a significant decline in fruity note (*p* < 0.05). Adding medium (680 mg/L) and low concentrations (66 mg/L) of isoborneol to the FR raised the woody strength (*p* < 0.01). Adding medium(680 mg/L) of isoborneol to the TR increased the overall aroma intensity (*p* < 0.01).And adding high concentration of isoborneol (3065 mg/L) led to a remarkably increase in herbal notes(*p* < 0.001).Only the isoborneol at the high level was added to TR had significant effects on green note (*p* < 0.001), and no significantly when TR in alkanes was supplemented with isoborneol at middle or low concentrations.It is noteworthy that when TR in alkanes was supplemented with the isoborneol at the low level, there was no significant effect on the overall aroma intensity, but it has a significant effect on the woody note (*p* < 0.01), which changed the aroma structure.There is no significant effect on the floral note when TR in alkanes was supplemented with isoborneol at any concentrations.

#### 3.3.2. Modulation of Sensory Attributes by Terpene Alcohols Addition

**Figure 6:**
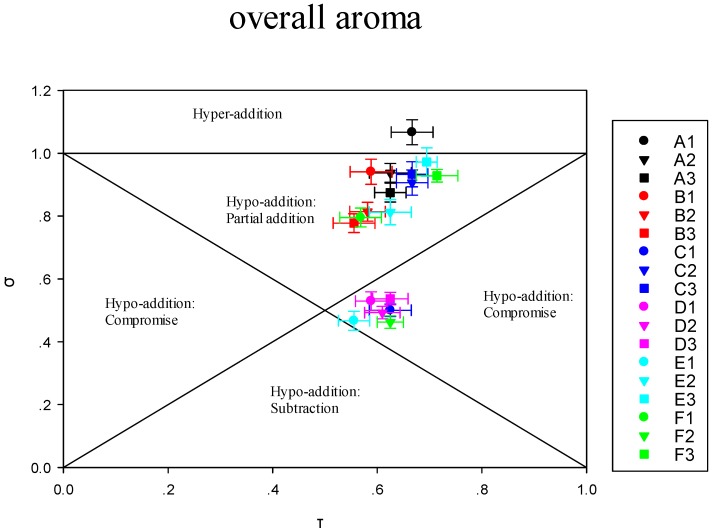

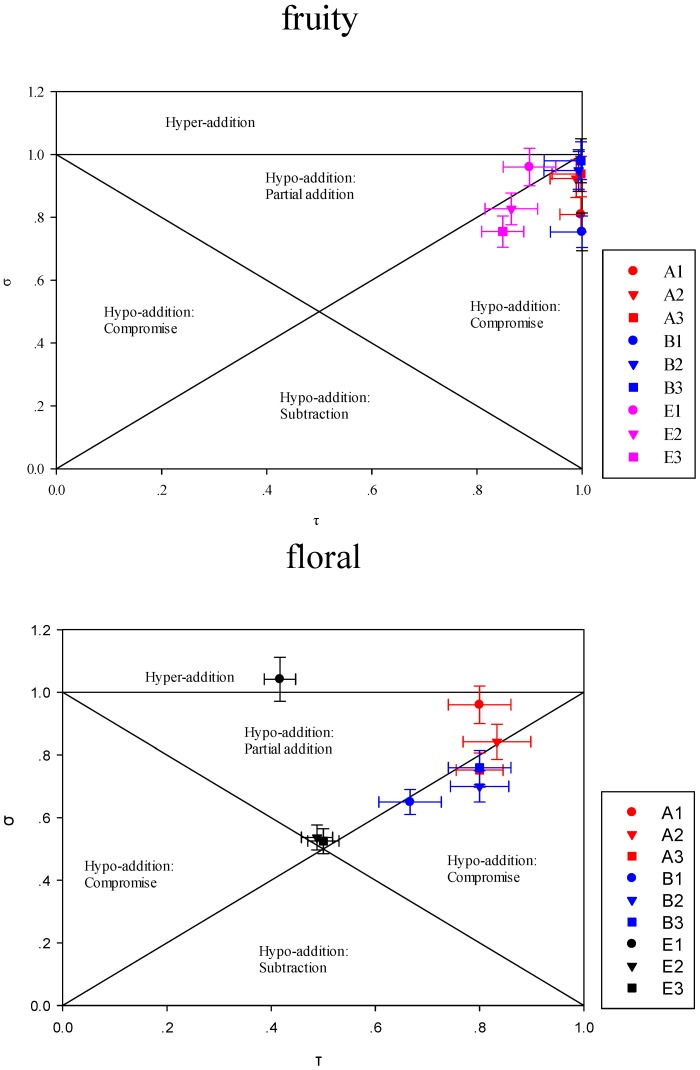

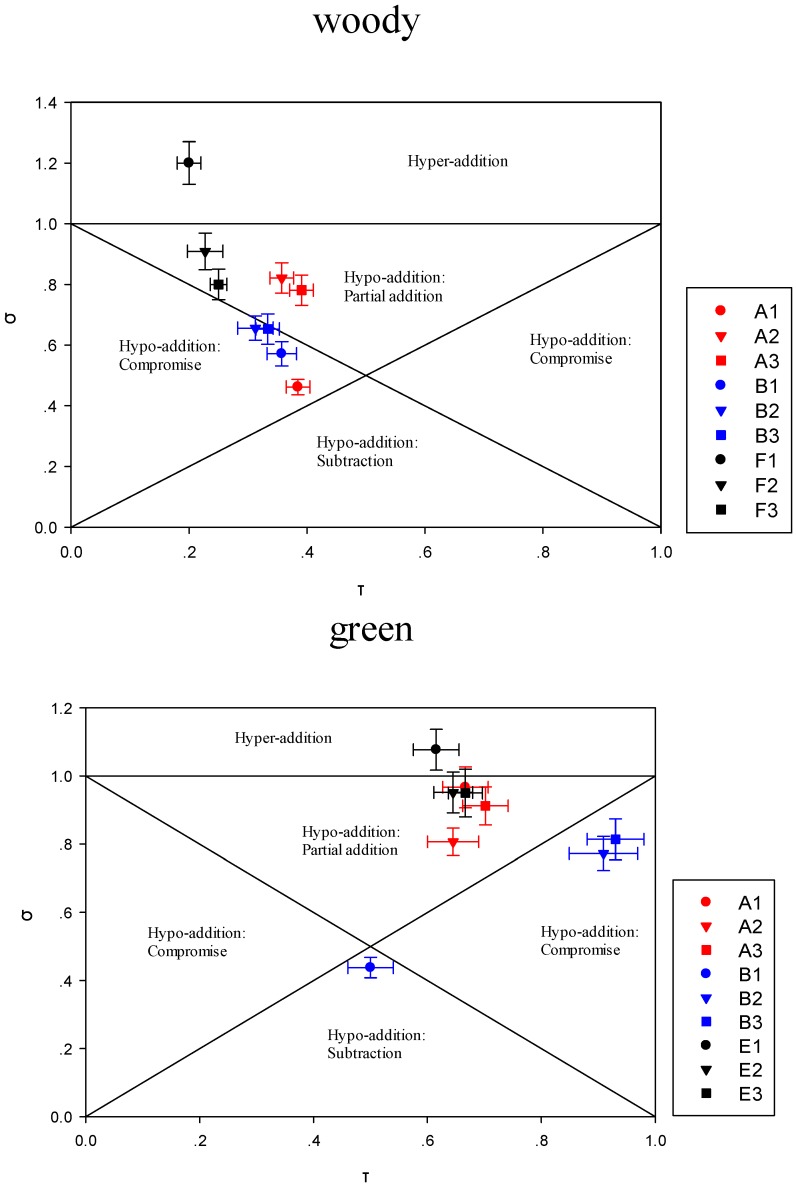

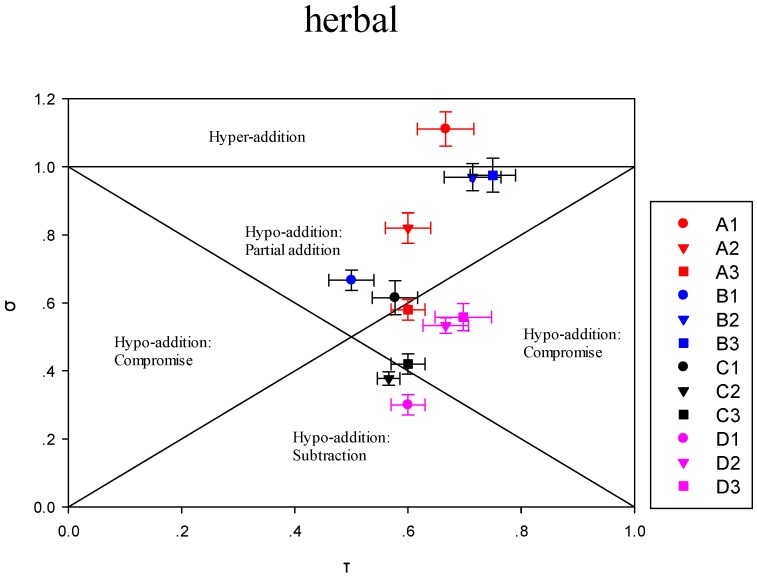
Individual impact of alcohols on sensory attributes of Terpenes Recombination. 1–3, at high, medium, and low level respectively. A, isoborneol; B, d-fenchyl alcohol; C, mixture of borneol and eudesmol; D, mixture of borneol and linalool; E, mixture of eudesmol and linalool; and F, mixture of borneol, eudesmol and linalool.

In this research, formula σ = f(τ) was used to analyze the quantitative olfactory interactions. Cain and Drexler [17] mentioned the judgment of the mixture interaction was to compare the strength of the mixture with the strength of each individual component. Frijters [43] distinguished between three hypoaddition cases: The terms “partial addition”, “compromise”, and “subtraction”. Addition of isoborneol to FR in alkane solution led to an addition effect on overall aroma in any levels of concentrations (Figure 6). Adding BE at high level (C1) to TR has compromised the overall aroma, this conclusion is agreed with the results obtained by psychometric curve on the basis of Feller’s additive model.

As shown in Figure 6, adding EL with high concentration (E1) to TR in alkane solution led to hypo-addition of fruity note. Adding isoborneol (A1, A2, A3) and d-fenchyl alcohol (B1, B2, B3) led to compromise of fruity note. Studies have shown that compounds that are nonfruity aroma could affect the fruity note of TR. For instance, adding diacetyl, acetoin, acetic acid, and γ-butyrolactone had compromise effects on fruity note of Higher Alcohols [44]. Adding EL with high concentration (E1) to TR resulted in hyper-addition of floral note. For woody notes, adding BEL with high concentrations (F1) to TR resulted in hyper-addition. And adding d-fenchyl alcohol (B1, B2, B3) resulted in compromise. Adding EL with high concentration (E1) results in hyper-addition of green note, and adding isoborneol (A1, A2, A3) has a partial addition. When isoborneol (A1) was added simultaneously to TR in alkane solution, hyper-addition effect on the herbal descriptor was detected at high concentration. More precisely, adding BE, BL at high concentration (C1, D1) have resulted in a subtraction effect on herbal note. More importantly, in terms of those aroma impacts on perception, this was consistent with the result of the sensory evaluation.

### 3.4. The Contribution of Isoborneol to Overall Flavor

#### 3.4.1. Sensory Analysis

**Figure 7:**
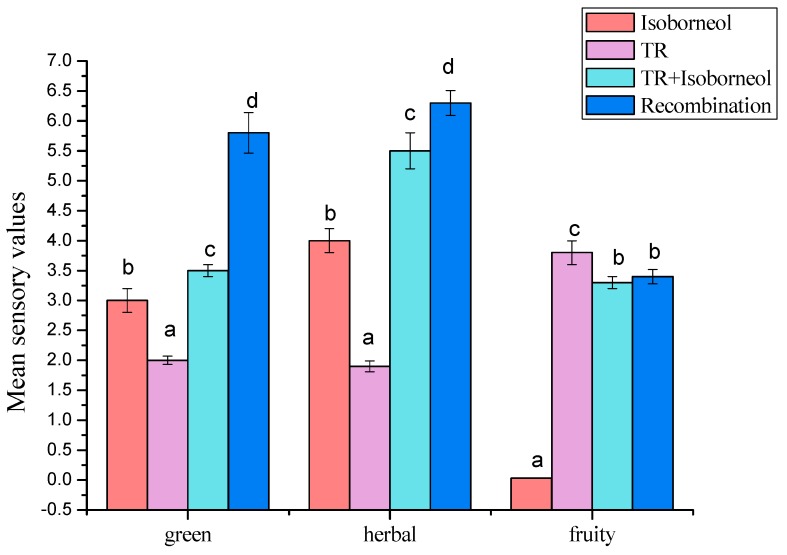
Results of sensory analysis of four samples.

As shown in Figure 7, the sensory attributes of recombination, isoborneol, TR, and the mixture of isoborneol and TR were described as green, herbal, and fruity notes. One Way Analysis of Variance was employed to distinguish statistical differences among the aromatic recombinations, in view of three replicates of sensory assessment. The result showed diverse degrees of differences in the attributes (*p* < 0.05). Tukey’s tests indicated that herbal and green notes had significant differences in different concentrations for each sample. The mixture of isoborneol and TR was significantly different from the TR alone in all attributes.

As presented in Figure 7, recombination had the highest score in green and herbal notes. However, the variation was not apparent in fruity note. This result also verified the conclusion of Figure 5. When isoborneol and TR was mixed, the mixture showed higher herbal note than isoborneol and TR. A similar situation was noted in green attribute. In the addition, the mixture showed a lower fruity note. Consequently, when isoborneol and TR were mixed, the characteristic aromas like herbal and green notes were enhanced and the fruity note was reduced.

#### 3.4.2. Electronic Nose Analysis

The electronic nose is able to detect the volatile profile of a mixture without separating volatiles, and has proven to be promising for aroma evaluation [45,46]. It was applied to estimate the effect of isoborneolon terpenes recombination odor by the aroma volatile profile in this experiment.

**Figure 8:**
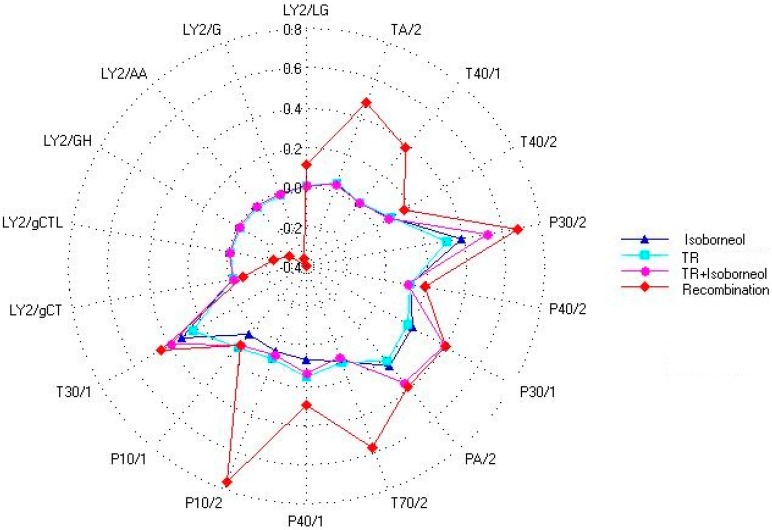
Radar Graph of four samples by e-nose.

The samples of recombination, isoborneol, TR and the mixture of isoborneol, and TR were analyzed by e-nose, a radar graph (Figure 8) was built in Excel. Isoborneol has herbal and green notes in the literature. And the sensory analyses show the same results. According to Figure 8, the parameters of the 18 sensors can clearly distinguish the difference between the recombination and the other three samples. The aroma profiles of isoborneol and TR, were different in P30/2, P30/1, P40/1, P10/1, and T30/1. In addition, compared with isoborneol sample and TR sample, the mixture of them showed higher responding signals in sensors P30/1, P30/2, PA/2, and T30/1. The responding values of mixture were very close to those of recombination sample on sensors LY2/LG, P30/1, T40/2, PA/2, T30/1, and P10/1. Thus, it is indicated that the aroma of isoborneol and TR after being mixed had changed and it was closer to the overall flavor in some sensors, which agreed with the results obtained from previous olfactory evaluation.

#### 3.4.3. Relationship between Aromatic Recombinations, E-Nose Data, and Olfactory Descriptors

**Figure 9:**
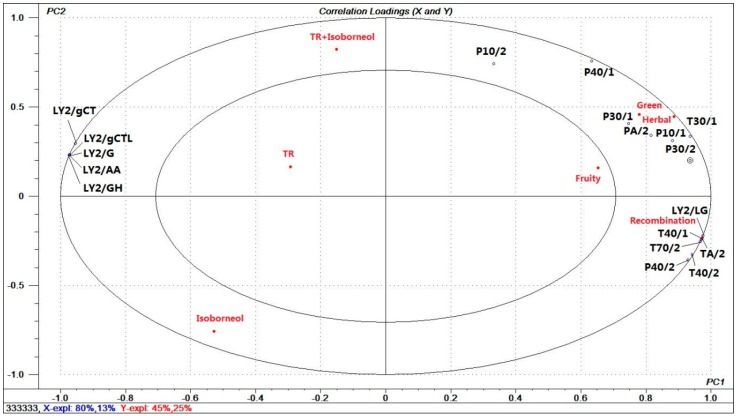
Relation diagram of partial least-squares regression.

PLSR was used to process the mean values of the scores accumulated from sensory evaluation by the sensors response values, sensory descriptors and samples. The X-matrix represented the response values of these sensors. The Y-matrix represented sensory variables and samples. The result can be seen from Figure 9. The PLSR model explained 93% two-factor model of the cross-validated variables. Figure 9 was showed as correlation loadings plot. Seven Y variables (recombination, isoborneol, TR, the mixture of isoborneol and TR, green, herbal, and fruity) and eighteen X variables were situated between the inner and outer ellipses, demonstrating that all of them were well explained by the PLSR model.

From Figure 9, we learned that isoborneol sample was situated at the negative area of PC1 and PC2. The mixture of isoborneol and TR, TR sample were located in the positive area of PC2 and the negative area of PC1. It is indicated a variation after mixing. Additionally, fruity was weak note in the inner ellipses, green and herbal notes have high explained variances between the inner and outer ellipses. Moreover, green and herbal descriptors were located in the first quadrant. It was attributed to PA/2, P30/1, T30/1, P30/2, and P10/1. In e-nose analysis, sensors P30/2, P30/1, PA/2, and T30/1 showed relatively significant changes in analysis between compounds before and after being mixed. It is indicated those sensors was correlated with green and herbal notes. Any sensors response values did not correlate with fruity attribute, which is in agreement with the sensory evaluation results. We can conclude that changes in some of the sensor parameters reflect changes in aromas and that these laws can be used to build aroma prediction models.

## 4. Conclusions

The addition of isoborneol at different levels found in Chrysanthemum essential oils revealed the interesting behavior. Isoborneol decreased the olfactory threshold of terpenes recombination and changed its perceptual expression, especially the intensities of herbal and fruity notes. Generally, it resulted in a compromise effect on fruity note and a partial addition on green and herbal notes in alkane solution when adding isoborneol.

Furthermore, adding terpene alcohols with different concentrations, leading to the diverse olfactory expression of the terpenes recombination. This discovery could herald the importance of alcohols in Chrysanthemum essential oils, during deploying the flavor fragrance, thus, although the interaction mechanism between alcohols and TR was complicated, we believe that the findings in this article have inspired the aroma deployment process. This article hopes to use these basic data in combination with electronic nose technology to establish a model for evaluating aroma quality later, and can be a more efficient and accurate evaluation of aroma quality. Finally, it will be applied to food, cosmetics, tobacco, and other related industries.

## Figures and Tables

**Table 1:** Terpenes Concentrations Used for Sensory Analyses.

Terpenes	Concentration (mg/L)
α-pinene	3083
camphene	2780
β-pinene	355
β-myrcene	787
α-phellandrene	523
dl-limonene	8192
cis-ocimene	53
α-terpinolen	7566
caryophyllene	775
β-farnesene	243
Germacrene B	985
**alcohols**	
linalool	2974
d-fenchyl alcohol	1178
eudesmol	1177
borneol	183
isoborneol	3065
4-terpineol	866

**Table 2:** Amount of Alcohols Added to Aromatic Recombination for Sensory Profiles.

	L	F	E	B	I
high concentration (mg/L)	2974	1178	1177	183	3065
Medium concentration (mg/L)	1442	822	407	62	680
low concentration (mg/L)	81	123	13	13	66

L, linalool; F, d-fenchyl alcohol; E, eudesmol; B, borneol; I, isoborneol.

**Table 3:** Sensory effect of the addition of alcohols in terpenes recombination.

	13 Terpenes	L	F	E	B	I	T	Difference Observed
TR	e	-	-	-	-	-	-	
test 1	e	e	-	-	-	-	-	=
test 2	e	-	e	-	-	-	-	***
test 3	e	-	-	e	-	-	-	=
test 4	e	-	-	-	e	-	-	=
test 5	e	-	-	-	-	e	-	***
test 6	e	-	-	-	-	-	e	=
test 7	e	e	e	-	-	-	-	=
test 8	e	e	-	e	-	-	-	***
test 9	e	e	-	-	e	-	-	***
test 10	e	e	-	-	-	e	-	=
test 11	e	e	-	-	-	-	e	=
test 12	e	-	e	e	-	-	-	=
test 13	e	-	e	-	e	-	-	=
test 14	e	-	e	-	-	e	-	=
test 15	e	-	e	-	-	-	e	=
test 16	e	-	-	e	e	-	-	***
test 17	e	-	-	e	-	e	-	=
test 18	e	-	-	e	-	-	e	=
test 19	e	-	-	-	e	e	-	=
test 20	e	-	-	-	e	-	e	=
test 21	e	-	-	-	-	e	e	=
test 22	e	e	e	e	-	e	-	**
test 23	e	e	e	e	e	e	-	*
test 24	e	e	e	e	e	-	e	**
test 25	e	e	e	e	-	e	e	*
test 26	e	e	-	e	e	e	e	**

***, the significance level is 0.1%; **, the significance level is 1%; *, the significance level is 5%; =, no significance level; e, existence of compound; -, lack of compound; L, linalool; F, d-fenchyl alcohol; E, eudesmol; B, borneol; I, isoborneol; and T, 4-terpineol.

**Table 4:** Omission tests of alcohols from the complete recombination.

	13 Terpenes	L	F	E	B	I	T	Difference Observed
CR	e	e	e	e	e	e	e	
test 27	e	-	e	e	e	e	e	=
test 28	e	e	-	e	e	e	e	*
test 29	e	e	e	-	e	e	e	=
test 30	e	e	e	e	-	e	e	=
test 31	e	e	e	e	e	-	e	***
test 32	e	e	e	e	e	e	-	=
test 33	e	-	-	e	e	e	e	=
test 34	e	-	e	-	e	e	e	*
test 35	e	-	e	e	-	e	e	*
test 36	e	-	e	e	e	-	e	***
test 37	e	-	e	e	e	e	-	=
test 38	e	e	-	-	e	e	e	=
test 39	e	e	-	e	-	e	e	=
test 40	e	e	-	e	e	-	e	***
test 41	e	e	-	e	e	e	-	=
test 42	e	e	e	-	-	e	e	**
test 43	e	e	e	-	e	-	e	***
test 44	e	e	e	-	e	e	-	=
test 45	e	e	e	e	-	-	e	***
test 46	e	e	e	e	-	e	-	=
test 47	e	e	e	e	e	-	-	***
test 48	e	-	-	e	e	-	e	***
test 49	e	-	e	-	e	-	e	***
test 50	e	-	e	-	e	-	e	***
test 51	e	-	e	e	-	-	e	***
test 52	e	-	e	e	e	-	-	***
test 53	e	e	-	-	e	-	e	***
test 54	e	e	-	e	-	-	e	***
test 55	e	e	-	e	e	-	-	***
test 56	e	-	-	e	-	-	e	***
test 57	e	-	-	e	e	-	-	***
test 58	e	-	e	-	-	-	-	***

***, the significance level is 0.1%; **, the significance level is 1%; *, the significance level is 5%; =, no significance level; e, existence of compound; -, lack of compound; L, linalool; F, d-fenchyl alcohol; E, eudesmol; B, borneol; I, isoborneol; and T, 4-terpineol.

**Table 5:** Olfactory Threshold of aroma compounds in alkane Solution and TR Solution.

Sample	Diluted Concentration (mg/L)	C	D	R	OT (mg/L)
	in alkanes Solution				
I	22/11/5.50/2.75/1.88/0.94/0.47/0.24/0.12/0.06	−0.216	0.267	0.964	0.61
F	380/190/95/47.50/23.75/11.88/5.94/2.97/1.48/0.74	1.096	0.367	0.985	12.47
B	84/42/21/10.50/5.25/2.63/1.82/0.91/0.46/0.23	0.117	0.433	0.991	2.03
E	3120/1560/780/390/195/97.50/48.75/24.38/12.19/6.10	1.991	0.218	0.992	97.95
L	460/230/115/57.50/28.75/14.38/7.19/3.60/1.80/0.90	0.585	0.367	0.980	3.85
BE	145/72.50/36.25/18.13/9.07/4.53/2.27/1.13/0.57/0.28	0.190	0.424	0.979	1.55
BL	2.89/1.45/0.72/0.36/0.18/0.09/0.05/0.02/0.01/0.006	−1.284	0.445	0.970	0.05
EL	6.25/3.13/1.57/0.79/0.40/0.20/0.10/0.05/0.03/0.01	−1.088	0.604	0.957	0.08
BEL	88/44/22/11/5.50/2.75/1.88/0.94/0.47/0.24	−0.060	0.536	0.954	0.87
TR	800/400/200/100/50/25/12.5/6.25/3.13/1.57	1.487	0.388	0.978	30.69
	in TR Solution				
I	100/50/25/12.5/6.25/3.13/1.57/0.79/0.39/0.20	0.507	0.488	0.986	3.22
F	500/250/125/62.50/31.25/15.63/7.81/3.91/1.95/0.98	1.105	0.469	0.979	12.74
BE	3400/1700/850/425/212.50/106.25/53.13/26.56/13.28/6.64	1.841	0.482	0.986	69.34
BL	280/140/70/35/17.50/8.75/4.38/2.19/1.09/0.55	0.944	0.440	0.976	8.79
EL	928/464/232/116/58/29/14.50/7.25/3.63/1.81	1.335	0.294	0.989	21.63

L, linalool; F, d-fenchyl alcohol; E, eudesmol; B, borneol; I, isoborneol; BE, mixture of borneol and eudesmol; BL, mixture of borneol and linalool; EL, mixture of eudesmol and linalool; BEL, mixture of borneol, eudesmol and linalool; TR, Terpenes Recombination; C, the olfactory threshold taking mg/L as a unit; D, the slope of the function; R, fitting degree; OT, the olfactory threshold of the aromatic compound.
